# A harbinger of plate tectonics: a commentary on Bullard, Everett and Smith (1965) ‘The fit of the continents around the Atlantic’

**DOI:** 10.1098/rsta.2014.0227

**Published:** 2015-04-13

**Authors:** John F. Dewey

**Affiliations:** University College, Oxford OX1 5NA, UK

**Keywords:** continental drift, continental fitting, plate tectonics

## Abstract

In the 1960s, geology was transformed by the paradigm of plate tectonics. The 1965 paper of Bullard, Everett and Smith was a linking transition between the theories of continental drift and plate tectonics. They showed, conclusively, that the continents around the Atlantic were once contiguous and that the Atlantic Ocean had grown at rates of a few centimetres per year since the Early Jurassic, about 160 Ma. They achieved fits of the continental margins at the 500 fathom line (approx. 900 m), not the shorelines, by minimizing misfits between conjugate margins and finding axes, poles and angles of rotation, using Euler's theorem, that defined the unique single finite difference rotation that carried congruent continents from contiguity to their present positions, recognizing that the real motion may have been more complex around a number of finite motion poles. Critically, they were concerned only with kinematic reality and were not restricted by considerations of the mechanism by which continents split and oceans grow. Many of the defining features of plate tectonics were explicit or implicit in their reconstructions, such as the torsional rigidity of continents, Euler's theorem, closure of the Tethyan ocean(s), major continental margin shear zones, the rapid rotation of small continental blocks (Iberia) around nearby poles, the consequent opening of small wedge-shaped oceans (Bay of Biscay), and misfit overlaps (deltas and volcanic piles) and underlaps (stretched continental edges). This commentary was written to celebrate the 350th anniversary of the journal *Philosophical Transactions of the Royal Society*.

## 1. The pre-1965 harbingers

The general similarity of the shapes of the eastern and western shorelines of South America and Africa, respectively, was noticed and recorded by Francis Bacon at a time when the detailed geography of the shorelines was ill-determined and by Antonin Snider [1] when the existence of the continental shelf and its margin were unknown. Bacon did not claim or even mention that this implied that Africa and South America once fitted together. Alfred Wegener [2] and Alexander Du Toit [3] proposed the idea of continental drift and the opening of the South Atlantic from the excellent geometric fit of the South Atlantic continental margins (the edge of the continental shelf not the shorelines), the close similarity of South American Upper Palaeozoic faunas and floras, and the continuity of geological zones and structures when the two continents were restored to contiguity. Implicit but not explicit was that the two continental margins and, by inference, the continents must have been torsionally rigid to maintain the fitting edge. In retrospect, this seems to be compelling evidence for continental drift yet it was a subject for lively, often vituperative, debate until the 1960s. There was substantial division between geologists working in the Southern Hemisphere, where the evidence for continental drift is so clear, and the Northern Hemisphere where the evidence is less clear. Lester King (e.g. [4]), of the University of Cape Town, travelled the globe after World War II proselytizing for continental drift. As an undergraduate in London in 1956, I heard him talk in Imperial College, clearly and convincingly, when I became convinced that continental drift must be a reality. Stanley Hollingworth and Stanley Westoll, professors of geology at University College London and Newcastle, respectively, were firm adherents of drift in the 1950s. Westoll enjoyed taking a sheet of newsprint, tearing it along a jagged edge and then defying onlookers to claim that the fit of the two margins was a coincidence, a simple but salutary lesson to the anti-drifters. The conflict was exacerbated by Harold Jeffreys [5,6], who argued that continental drift is impossible from the theoretical standpoint that there was no known mechanism to explain it. This was a classic case not only of theory outrunning observation but theory constructed upon the incorrect notion that weak continents, dominated by the strength of quartz, ploughed through a strong oceanic basement dominated by the strength of olivine. Jeffreys' assumptions were incorrect but his influence was profound until the mid-1960s, especially in the USA. It is said that, if one espoused the ideas of continental drift in the USA before the 1960s, an academic career in geology was doomed. From 1970, the reverse has been true. Major figures of great influence such as Walter Bucher, Charles Drake and Maurice Ewing strongly opposed continental drift, the last until his death in 1974. Meyerhoff [7] waged warfare against plate tectonics to his death in 1994. As a young lecturer in Cambridge in the late 1960s, Jeffreys and R. A. Lyttleton, an expanding Earth enthusiast, suggested that I abandon the mobilist ‘nonsense’ that was gaining ground and credence because it would ruin my career. Opposition to mobilism could be construed as strange in that Keith Runcorn [8] and Ted Irving [9] had already demonstrated, with great precision and clarity, the relative motion of continents from palaeomagnetic studies.

All this, until the 1960s, was concerned with continental edge fitting because the geology of the ocean floor was poorly known, although Arthur Holmes [10] and David Griggs [11] had proposed a convective model involving ocean growth and destruction. By the mid-1960s, the geology of the ocean floor was becoming better known and the concept of sea-floor spreading along the axes of mid-ocean ridges and subduction in oceanic trenches had been proposed by Bob Dietz [12] and Harry Hess [13]. This, with the explanation of Vine & Matthews [14] of the zebra-like stripes of positive and negative magnetic anomalies as a ‘sea-floor spreading magnetic tape recorder’, laid much of the basis for plate tectonics. ‘Sam’ Warren Carey [15,16] developed several important contributions to ‘mobilism’ including the fast rotation of micro-continents and the opening of oceanic sphenochasms such as Iberia and the Bay of Biscay, and the growth of nemataths (hotspot tracks). Carey, like Bruce Heezen [17], failed to embrace and contribute to the plate tectonics revolution by hoisting his flag upon the mast of an expanding Earth, in spite of the clear evidence, from palaeomagnetic triangulation, of a constant size Earth at least during the Phanerozoic. By the mid-1960s, the contrast between old silicic/intermediate continents and a young mafic/ultramafic ocean floor was becoming clear.

A little known but profoundly prescient and original harbinger of the concept of a rotation pole that describes the relative motion of continental masses was Quennell [18]. Quennell recognized that the general trend of the Dead Sea fault system forms part of a small circle, normal to which great circles intersect at a pole near Gibraltar. From this, Quennell argued that the relative motion of Arabia with respect to Africa can be described as an anticlockwise rotation whose rate is given by the slip-rate (about 20 mm yr^−1^) on the sinistral Dead Sea Fault, now termed a transform fault, which also accounts for the opening of the Red Sea. This was a remarkable discovery of the use of a rotation pole to describe the relative motion of torsionally rigid continents. Another related idea before its time was Wellman's [19] recognition of the role of the Alpine fault in New Zealand as a dextral, crustal scale strike-slip fault that transferred motion between two zones of convergence, now termed a trench–trench transform.

Tuzo Wilson entered, enthusiastically, the mobilism saga in the early 1960s, before which he had been a fixist in the North American tradition. His first key paper [20] compared the Cabot Fault of the Maritime Provinces of Canada with the Great Glen Fault of Scotland supporting the opening of the North Atlantic. Similarly, Dewey & Kay [21] showed, in a reconstruction of the North Atlantic, the clear continuity of the Appalachian and Caledonian Belts. Wilson [22] used Carey's nemataths as hotspot track lines to demonstrate the movement of the ocean floor across plumes in the mantle and the relative motion of the ocean floor with respect to oceanic ridges. Especially persuasive is the V-shaped relationship of the Rio Grande Rise and Walvis Ridge to the Tristan da Cunha Hotspot. Wilson [23] not only explained the transform offsets of ridge axes and subduction zones, but also laid out a comprehensive global model of sea-floor spreading, major strike-slip and subduction along a network of connected earthquake boundaries, showing how transforms that connect ridges and subduction zones and subduction zones with different polarities cause the earthquake-bounded areas (plates) to change size and shape. Tuzo Wilson was, tectonically, almost there. In the early 1970s, he confided to me and others that, had he known of Euler's theorem, he would have nailed the theory of plate tectonics. Like Wegener, Du Toit and Carey, the torsional rigidity of ‘plates’ was implicit in his 1963 and 1965 papers, but he missed the essential point of the relative motion of plates described by rotations around axes passing through the centre of Earth intersecting the surface of Earth at poles of rotation. The scene was now set for Bullard *et al*. [24], which has become the central and most durable paper from a 1964 Royal Society Discussion Meeting on continental drift organized by Blackett, Bullard and Runcorn. The volume, published in 1965 [25], provides a fascinating snapshot of the point at which majority opinion was poised to swing from ‘fixist’ to ‘mobilist’ interpretations of oceans and continents, prominently driven by the rapidly increasing quality of palaeomagnetic data.

## 2. Bullard, Everett and Smith, 1965

Bullard *et al.* [24] was the key paper in the paradigm change that led from continental drift to plate tectonics. The essential, critical and original features of the paper are as follows:

1. The complete separation of the kinematic certainty of continental drift from concerns about the mechanism by which it was achieved.
2. Restoration of the continents around the Atlantic was achieved by minimizing the misfit between the true continental margins (edge of the continental shelf at the 50 fathom line, about 900 m) not the shorelines. Snug and convincing fits were achieved.
3. Torsional rigidity of the continents was assumed because distortion of continents and their margins would prohibit fitting. The superb fit demonstrates torsional rigidity.
4. Fitting of continental margins was done by using finite difference angular rotations around axes passing through the centre of Earth intersecting Earth's surface at poles of rotation. Euler's theorem was used to make the finite difference rotations.
5. Rifted continental margins may be very sharp with a rapid transition from continent to ocean, the ideal margin that fits cleanly. Overlaps were explained mainly as the growth of deltas since continental separation and voluminous basaltic lava fields.
6. Underlaps are mainly the result of continental stretching rather than a sharp break.
7. The recognition that smaller complicated areas like the Caribbean and Gulf of Mexico need special geological analyses to unravel the behaviour of small blocks.
8. When the continents around the Atlantic are restored to a ‘Pangea configuration’ (all continents fitted together), there appears the huge, westward-narrowing and terminating gap of the Tethyan ocean complex, which closed as a result of the Central and North Atlantic opening at different times around different poles of rotation.
9. Whereas most Atlantic margins were the site of extensional rifting, several, such as the equatorial Atlantic and the boundary between Greenland and Svaalbard, enjoyed hundreds of kilometres of strike-slip relative motion along giant intracontinental transform faults.
10. Some small continental blocks, such as Iberia, have rotated by up to 40^°^ during periods as short as 10 Myr around nearby rotation poles to open small, wedge-shaped oceans (Carey's sphenochasms) such as the Bay of Biscay.

These were profoundly important reconstructions, results and conclusions that led directly to the formulation of the theory of plate tectonics shortly afterwards, and then to its geological implications and corollaries. Reception of the paper was enthusiastic and enhanced by the very high quality of the drafting of key figures in well-chosen geographical projections with publication in colour; overlap and gaps were shown, respectively, in red and blue. The fitting was achieved using a least-squares misfit between digitized continental margins on a sphere, which avoided the problems of earlier attempts that used moulded caps on a spherical last or simply ignored the issues of sphericity. Early fit diagrams were semi-schematic, whereas those of Bullard *et al*. [24] were based upon a simple and clear mathematical construction and a fitting algorithm on Earth's spherical surface based upon explicit rotations. The Earth science community saw the paper as a major breakthrough in tectonics; it was used widely in structure and tectonics courses, including mine, as an advance in our quantitative understanding of tectonics. It led to similar studies using the same techniques (e.g. [26–28]).

## 3. Plate tectonics

Plate tectonics was formulated by McKenzie & Parker [29]; they showed how the tectonics of the North Pacific can be described, very simply and elegantly, by the relative motion of a small number of torsionally rigid lithospheric plates (Pacific, Juan de Fuca, North America around poles of rotation). Three basic types of plate boundary were recognized: ridges where new oceanic lithosphere is created; trenches where lithosphere is subducted; and transform faults where the relative motion is accommodated along lithosphere-penetrating transcurrent faults. This paper transformed tectonics; within a few years, dozens of papers elaborated many aspects of plate tectonics. McKenzie [30] speculated on the consequences and causes of relative plate motion and McKenzie & Morgan [31] wrote the definitive paper on plate triple junctions. Sykes [32] supported Wilson's hypothesis of ridge to ridge transforms using earthquake first motions. Comprehensive global plate tectonic models were developed by Morgan [33], Isacks *et al*. [34] and Le Pichon [35]. Error-minimizing global vector nests for instantaneous relative plate rotations were developed by Chase [36], Minster *et al.* [37] and De Mets [38]; these provided, for the first time, a comprehensive quantitative solution for the relative motion of the global plate system.

Plate tectonics had an immediate and massive effect upon the geological world in a way that continental drift never had. Plate tectonics provided the basis for understanding most of tectonic and historical geology (e.g. [39–42]) such as rift valleys, mountain belts, major strike-slip faults, deep ocean trenches, volcanic arcs and the evolution of the continental crust and large regions of Earth. Essentially, this was the qualitative geological ‘clothing’ for plate tectonics. The term ‘Wilson cycle’ was adopted for the opening and closing of oceans from continental splitting through arc–continent collision, to continent–continent collision in honour of his paper that first explained the Appalachian–Caledonian Orogen in terms of ocean opening and closing [43]. It was soon realized, from earthquake distribution, that plate boundaries in oceans are generally very sharp and well defined in contrast to the more complicated and diffuse plate boundary zones of continents. Soon after the oceanic lithosphere is generated by sea-floor spreading, it acquires great torsional rigidity.

Of especial importance was the development of the quantitative relationship between plate tectonics and geology. The first paper of this genre was Atwater [44] in which the basic tectonic evolution of the US Cordillera was related to the relative motion of the Cordilleran margin to the plates of the eastern Pacific. Smith [45] and Dewey *et al.* [28] related the opening of the Central and North Atlantic around different poles of rotation to the closing of the Tethyan oceanic ‘gap’ and the development of plate boundaries and tectonics in the Alpine orogenic system. Sengor [46] introduced the concepts of Palaeo-Tethys, the larger ocean(s) that formed the gap on the Bullard *et al.* reconstruction, and Neo-Tethys oceans that were formed by the detachment of continental strips during the closure of Palaeo-Tethys. Pindell & Dewey [47] described the tectonic evolution of the Gulf of Mexico and Caribbean region by analysing the history of the relative motion of North and South America. Dewey [48] provided a basic framework for the complex changing evolution of plate boundary zone rock systems from the inherent complexities of plate boundary evolution. He showed that poles of rotation are of three types: finite difference (Euler poles), finite motion and instantaneous. In a three-plate system, only two poles can be finite motion; the third must be only instantaneous with respect to which its plate boundary must move. In other words, a three-plate vector triangle cannot close except instantaneously at a point. It is likely that most poles are only instantaneous.

## 4. Further implications

**Figure 1.**
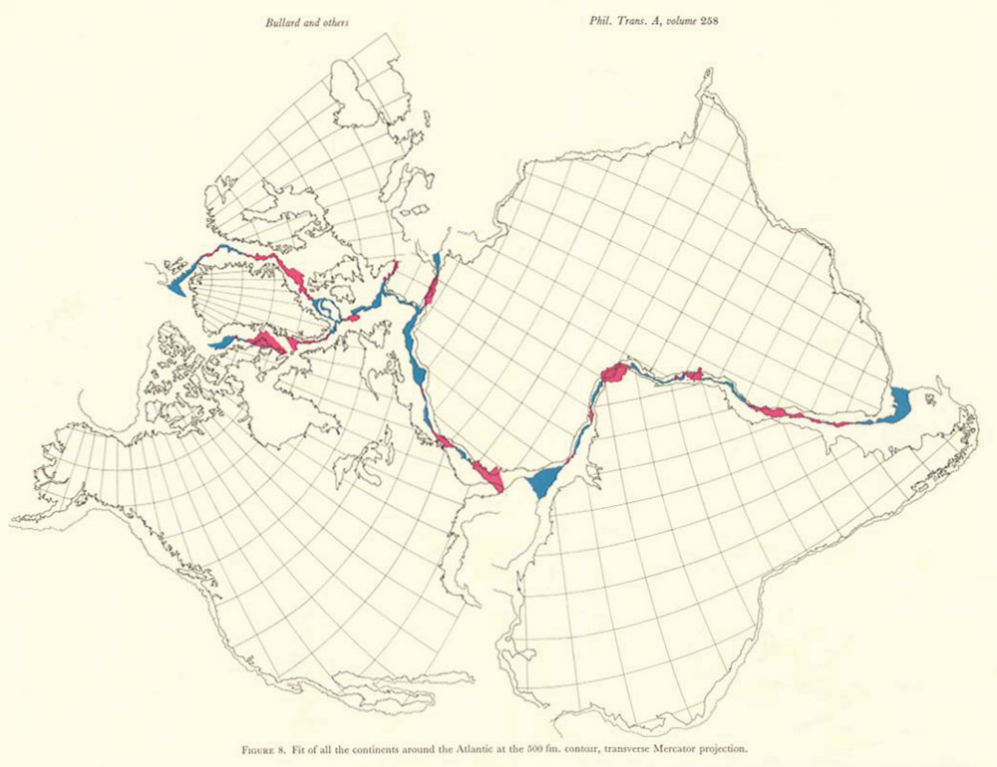
The fit of the continents around the Atlantic [24]. Copyright The Royal Society.

The quantification of continental drift [24] leading to plate tectonics [29], in the context of the 1960s overall, was the greatest paradigm change in twentieth century geology. There are few areas of geology that it has not touched from tectonics to palaeontology; for the last 47 years, it has formed the basis for most of our understanding of Earth's evolution. There are, however, a substantial numbers of corollaries of plate tectonics of which we have less than a full picture and understanding. There is still disagreement about the rheology of the lithosphere, where its strength lies, how its strength varies, and the cause of its torsional rigidity [49,50]. Perhaps the biggest challenge that tectonic and structural geology faces is the detailed relationship between relative plate motion and rock structures and strain in continental plate boundary zones, and as a result of evolving plate boundaries across which the slip vector changes, and triple junctions that change their position and type (figure 1). Analyses have been attempted, fairly successfully, in transtensional zones between blocks with plate boundary zones such as the Eastern California Shear Zone, where Dewey & Taylor [51,52] used GPS, the inversion of large earthquake arrays, palaeomagnetism, volcanic systems and the detailed kinematics of fault systems to deduce the evolution of bulk strain facies patterns in area and depth in the Coso region. This approach can be conducted only in superbly exposed regions and involves a huge amount of field mapping and structural analysis. Also, as yet, it has been done only in a few regions at less than whole plate boundary zone scale. GPS has been a powerful tool in measuring instantaneous relative plate motion and relative block motions within continental plate boundary zones. Analysis of earthquakes alone gives a useful but incomplete picture because a substantial component of bulk displacement and strain occurs aseismically or accompanied by very small earthquakes. A further problem is why continents rift along older orogens, for example, the Greenland/Baltica split along the Caledonides and the Central Atlantic along the Appalachians/Mauritanides [53].

A final word about Teddy Bullard the man. He was a larger-than-life, wonderfully exuberant and enthusiastic person of great charm and friendliness. He was extraordinarily curious about everything and infected all those around him with a desire to find things out. As a young lecturer in Cambridge during the 1960s, he both enthused me and gave me profound advice, especially not to get stuck in a small niche of geology permanently but to expand the scale and range of one's research, to follow one's own interests not necessarily the flavour of the month and certainly not the proactive demands of senior figures, indeed any others, especially committees, who claim, falsely, to know what is important, and to always stay with science, rejecting arid administrative paths. His influence on the scientific world was profound. All who knew him will remember him with great affection for his wisdom, cleverness, originality, advice, guidance, friendship and the sheer fun of being with him.

## Author profile

**John Dewey** is Distinguished Professor Emeritus in the University of California and Fellow of University College, Oxford with visiting appointments at the Australian National University, the University of Texas at Austin and the British Museum of Natural History. His research interests include the growth and decay of mountain ranges, the origin, ascent and emplacement of granites, the kinematics of non-coaxial deformation zones and the origin and emplacement of ophiolite complexes. Most of his research is strongly field-based in Ireland, Scotland, Newfoudland, California, New Zealand and South Africa. As a lecturer in Cambridge during the plate tectonic revolution of the mid-to-late 1960s, he was a colleague of Alan Smith and became a personal friend of Sir Edward Bullard. In 1967 as plate tectonics burst upon the scene, he was fortunate to be taking a sabbatical in the Lamont Geological Observatory where much of the new mobilism was being played out.
